# Interactions between Core Elements of the *Botrytis cinerea* Circadian Clock Are Modulated by Light and Different Protein Domains

**DOI:** 10.3390/jof8050486

**Published:** 2022-05-06

**Authors:** Vicente Rojas, Francisco Salinas, Andrés Romero, Luis F. Larrondo, Paulo Canessa

**Affiliations:** 1 Departamento de Genética Molecular y Microbiología, Facultad de Ciencias Biológicas, Pontificia Universidad Católica de Chile, Santiago 8331150, Chile; vlrojas@uc.cl (V.R.); llarrondo@bio.puc.cl (L.F.L.); 2 ANID–Millennium Science Initiative–Millennium Institute for Integrative Biology (iBIO), Santiago 8331150, Chile; francisco.salinas@uach.cl (F.S.); andres.romero.q@ug.uchile.cl (A.R.); 3 Instituto de Bioquímica y Microbiología, Facultad de Ciencias, Universidad Austral de Chile, Valdivia 5090000, Chile; 4 Centro de Biotecnologia Vegetal, Facultad de Ciencias de la Vida, Universidad Andres Bello, Santiago 8370186, Chile

**Keywords:** *Botrytis cinerea*, photoreceptors, yeast, PAS and LOV domains, optogenetics

## Abstract

*Botrytis cinerea* possesses a complex light-sensing system composed of eleven photoreceptors. In *B. cinerea*, *bcwcl1* encodes for the BcWCL1 protein, the orthologue of the blue-light photoreceptor WC-1 from *Neurospora crassa*. The functional partner of BcWCL1 is the BcWCL2 protein, both interacting in the nucleus and forming the *B. cinerea* white collar complex (BcWCC). This complex is required for photomorphogenesis and circadian regulation. However, no molecular evidence shows a light-dependent interaction between the BcWCC components or light-sensing capabilities in BcWCL1. In this work, by employing a yeast two-hybrid system that allows for the *in vivo* analysis of protein–protein interactions, we confirm that BcWCL1 and BcWCL2 interact in the absence of light as well as upon blue-light stimulation, primarily through their PAS (Per-Arnt-Sim) domains. Deletion of the PAS domains present in BcWCL1 (BcWCL1^PAS∆^) or BcWCL2 (BcWCL2^PAS∆^) severely impairs the interaction between these proteins. Interestingly, the BcWCL1^PAS∆^ protein shows a blue-light response and interacts with BcWCL2 or BcWCL2^PAS∆^ upon light stimulation. Finally, we demonstrate that BcWCL1 and BcWCL1^PAS∆^ respond to blue light by introducing a point mutation in the photoactive cysteine, confirming that both proteins are capable of light sensing. Altogether, the results revealed the complexity of protein–protein interactions occurring between the core elements of the *B. cinerea* circadian clock.

## 1. Introduction

In filamentous fungi, light exerts multiple biological effects, such as eliciting protection mechanisms (e.g., against UV radiation), developmental processes including growth and photomorphogenic genetic programs (e.g., development of reproductive structures), and coordinating time-sensitive processes that are controlled by a circadian clock [1,2,3,4]. Nevertheless, most of what we know about fungal photobiology comes from the pioneer studies performed in the saprophytes *Neurospora crassa* and *Aspergillus nidulans* [3], with otherwise limited information regarding important plant pathogens such as *Botrytis cinerea* [5]. This ascomycete, an important biological model for understanding the necrotrophic mode of plant infection, is also a relevant microorganism for agronomy, considered the second fungal phytopathogen worldwide, infecting over 1000 economically important plants and crops [6,7].

Explained by the photosynthetic characteristics of plants, the light microenvironment provided by the leaves is significantly red-light-shifted and enriched in green light, also including UV and blue light [8]. Thus, the combination of transmitted, absorbed, and reflected light on the leaves’ surface creates a particular illuminated environment whose relevance for phytopathogens has not been analyzed in detail [9]. Interestingly, light perception in *B. cinerea* is not a new phenomenon. Almost fifty years ago, several investigations provided evidence on the morphological and developmental effects of broad-spectrum light on *B. cinerea* [10,11,12,13,14]. The *B. cinerea* genome encodes for eleven photoreceptors [5,15,16], which participate in distinct fungal developmental processes when subjected to different light wavelengths [17]. However, only a few of their respective loss-of-function mutants have been studied: *bcbop1*, *bcwcl1*, *bcphy3*, and *bccry1* & *2* [17,18,19,20].

Work performed on *N. crassa* several years ago [21] led to the characterization at the molecular level of fungal (blue) light perception that was initiated with the isolation of the photoreceptor WC-1 (white collar-1) [21,22]. By means of electrophoretic mobility shift assays (EMSAs), the authors showed that the gene product required for the light signal transduction pathway also binds to the promoter region of a blue-light-induced gene, behaving as a GATA-type zinc finger transcription factor (TF) containing a DNA binding domain (DBD) [21,22,23]. As expected from a TF/photoreceptor, the protein has a nuclear localization signal (NLS) to allow for the transcriptional regulation of gene expression and a light-oxygen and voltage (LOV) domain that binds flavin adenine dinucleotide (FAD) as a chromophore [23]. Upon the absorption of a photon, the chromophore induces conformational changes in the WC-1 protein conferring its photoreceptor activity. The LOV domain contains a critical cysteine residue to allow for the cysteinyl-flavin adduct formation [24]. Besides the NLS, LOV, and DBD domains, WC-1 also possesses two PAS (Per-Arnt-Sim) domains involved in protein–protein interactions, allowing for the formation of the white-collar complex (WCC) together with WC-2, another GATA-type TF [25]. Thus, the WCC serves a dual role, as it is a core component of the circadian oscillator, allowing for the rhythmic expression of the clock gene frequency (*frq*) and, on the other hand, it activates the transcriptional responses to blue light. 

The orthologs of the white-collar proteins in *B. cinerea*, BcWCL1 and BcWCL2, interact in the nucleus of the fungus, forming the *B. cinerea* white collar complex (BcWCC) [5,26]. The BcWCC, through BcWCL1, inhibits the conidiation required to display full virulence in the presence of light [17]. BcWCL1 is also required to deal effectively with oxidative stress and excessive light [17]. In addition, BcWCL1 is necessary for inducing several genes, such as *bcfrq1*, *bcvvd1*, and the great majority of photoreceptor-encoding genes [5,17]. As in *N. crassa*, the *B. cinerea frq* orthologue (*bcfrq1*), is pivotal for light entrainment of the circadian clock [17,27,28], while, for *bcvvd1*, the ortholog of the LOV-containing protein in *N. crassa* VIVID (VVD), there is no evidence of its role in photoadaptation [29,30,31]. Nonetheless, no molecular/experimental information has shown that the BcWCC function is (blue) light-dependent, nor how the BcWCL1 [17] and BcWCL2 interaction is modulated upon light.

To characterize the impact of (blue) light on the BcWCL1-BcWCL2 protein interaction, we took advantage of a new optogenetic platform developed in the budding yeast *Saccharomyces cerevisiae*, termed FUN-LOV [32]. Yeast can be considered an orthogonal “blind” chassis since its genome does not encode photoreceptors [33], providing a unique experimental system for analyzing these proteins. By employing the overall architecture of FUN-LOV and using different versions of BcWCL1 and BcWCL2 in a yeast two-hybrid molecular configuration, here, we show that the components of the *B. cinerea* WCC interact both in the absence of light as well as upon blue-light stimulation. Furthermore, we demonstrate that the PAS domains of BcWCL1 and BcWCL2 are necessary for their interaction in the absence or presence of (blue) light. Interestingly, the results also indicate that the LOV domain of BcWCL1 modulates the BcWCL1-BcWCL2 interaction in a light-dependent fashion, and that an unidentified region in BcWCL1 is also required for the interaction. Moreover, we show that the BcWCL1^PAS∆^ protein lacking both PAS domains retains the capacity to sense blue light. Altogether, the results suggest a complex dynamic of protein–protein interactions among core elements of the *B. cinerea* circadian clock.

## 2. Materials and Methods

### 2.1. Strains and Culture Conditions

The *S. cerevisiae* strain BY4741 with *GAL4* and *GAL80* deletions (*MATa*; *his3∆1*; *leu2∆0*; *met15∆0*; *ura3∆0*, *gal4∆::NatMx*, *gal80∆::HphMx*) was used as the genetic background for yeast transformation. This strain was maintained in YDPA medium (2% glucose, 2% peptone, 1% yeast extract, and 2% agar) at 30 °C. Co-transformants carrying plasmids with auxotrophic markers were maintained in synthetic complete (SC) media (0.67% yeast nitrogen base without amino acids, 2% glucose, 0.2% dropout mix, and 2% agar) minus the corresponding amino acid mixture (dropout mix).

### 2.2. In Silico Analysis of the Botrytis cinerea Proteins

The deduced proteins that form the positive arm of the *B. cinerea* circadian clock—BcWCL1 and BcWCL2 [27], as well as the photoreceptor BcVVD1, whose respective gene is light-induced [17]—were analyzed *in silico* to determine the presence of conserved protein domains, as well as the critical residues involved in light perception. For this purpose, the protein sequences were retrieved from the *B. cinerea* genome database [16], gene IDs: Bcin02g07400, Bcin05g05530, and Bcin13g01270, respectively, and analyzed with the InterPro Scan search tool [34]. The putative photoactive Cys residue within the LOV domain of BcWCL1 was identified using CLUSTAL Omega [35] employing the *N. crassa* WC-1 and VIVID proteins as references [36,37]. 

### 2.3. Plasmids and Genetic Constructs

The components of the original FUN-LOV system [32], such as the *ADH1* promoter, *ADH1* terminator, and the GAL4 DBD and AD domains, were used to assemble the genes of interest from *B. cinerea*. Importantly, we selected the *ADH1* promoter to command gene expression of the genetic constructs since we previously demonstrated its transcriptional activity during the exponential phase of yeast growth [32]. The genes encoding the full-length BcWCL1 and its version without both PAS domains (BcWCL1^PAS∆^) or the LOV domain (BcWCL1^LOV∆^) were synthetized using the Bio Basic Inc. gene synthesis service (ON, Canada). The BcWCL1 version solely carrying the protein region that contains the LOV domain (BcWCL1^LOV^; indicated in Table 1) was PCR amplified from the full-length version. All the genetic constructs carrying different variants of BcWCL1 were assembled with the Gal4-DBD and cloned into the pRS423 plasmid for *HIS3* auxotrophic selection. Similarly, the genes encoding the full-length version of BcWCL2 and its version without its PAS domain (BcWCL2^PAS∆^) were assembled with the GAL4-AD and cloned into pRS425 plasmid for *LEU2* auxotrophic selection. Deletion of the PAS domain of BcWCL2 was carried out by divergent PCR amplification and DNA gap repair in *Escherichia coli* [38]. All the genetic constructs were designed *in silico* and generated using yeast recombinational cloning (YRC) *in vivo* assembly [39]. Briefly, different DNA fragments were amplified using Phusion Flash High-Fidelity PCR Master Mix (Thermo Scientific, Waltham, MA, USA), employing oligonucleotides with 50 nt for direct YRC. Coding sequences of BcWCL1 and BcWCL2 were obtained from the B05.10 *B. cinerea* strain genome [16]. In the BcWCL1 and BcWCL1^PAS∆^ proteins, the photoactive cysteine (C414) was replaced by serine using site-directed mutagenesis [40]. Briefly, the template DNA encoding BcWCL1 or BcWCL1^PAS∆^ was PCR amplified using primers containing the mutation. After the PCR reaction, template DNA was digested with *Dpn*I and the PCR product was transformed into *E. coli* for DNA gap repair [38,40]. The primers used for plasmids assembly and site-directed mutagenesis are shown in Supplementary Table S1. The plasmids used and generated in this work are shown in Supplementary Table S2. All genetic constructs were sequenced on automatic sequencers employing fluorescent-based Sanger reactions (Macrogen Inc., Seoul, Korea).

### 2.4. Protein–Protein Interaction Assays

A destabilized luciferase reporter gene optimized for real-time monitoring of gene expression in yeast was used, as described [41]. The luciferase reporter gene was controlled by the *P_5XGAL1_* synthetic promoter [32], permitting luciferase expression upon the reconstitution of a two-hybrid system based on PAS-PAS or LOV-LOV interactions in the presence or absence of light [32]. The reporter gene expression levels were assayed under constant blue light (BL), constant darkness (DD), and a single BL pulse (BLP) of two-hour duration, using a custom LED illumination system recently described by [42] that provides blue light at 466 nm and applying 20 µmol m^2^ s^−1^ of light intensity. The measurements of optical density at 600 nm (OD_600nm_) and luminescence of the yeast cell cultures over time were simultaneously determined using a Cytation 3 or Synergy H1M microplate readers (BioTek, Winooski, VT, USA), which carry the same monochromator optical configuration. In all the experiments, yeast strains were grown overnight in a 96-well plate with 200 µL of SC medium at 30 °C in DD condition. Thereafter, 10 µL of these cultures was used to inoculate a new 96-well plate containing 190 µL of fresh media supplemented with 1 mM of luciferin [42]. This 96-well plate was incubated inside the plate reader for DD condition, where OD_600nm_ and the luminescence were acquired at 30 °C every 10 min and during 24 h, running high-resolution kinetic protocols with 30 sec of shaking before data acquisition [42]. In the BL and BLP conditions, the 96-well plate was incubated using a discontinuous kinetics protocol, maintaining the 96-well plate outside of the plate reader for illumination and inside of the equipment only for data acquisition [42]. The raw data of luciferase expression (luminescence) and OD_600nm_ for all the assayed experimental conditions are shown in Supplementary Figures S1–S3 and S5. The total amount of luciferase expression in the BLP condition was determined using the area under the luminescence curves according to [43]. The area under the luminescence curves was calculated using the GraphPad Prism Software version 9.3.1. All experiments were performed in six biological replicates. The yeast strains generated in this work are described in Supplementary Table S3. 

## 3. Results

### 3.1. The Components of the BcWCC Interact in the Presence or Absence of Light

**Figure 1:**
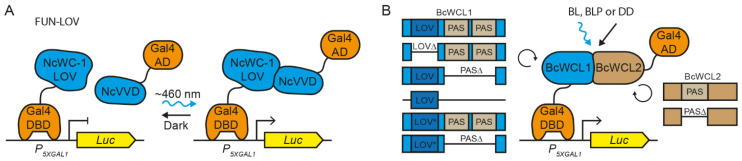
Protein–protein interaction assays performed for *B. cinerea* WCC proteins. (**A**) The FUN-LOV optogenetic switch architecture [32]. This system enables light-controlled gene expression in yeast through protein–protein interaction of the LOV domain of WC-1 and the photoreceptor VVD from *N. crassa*. (**B**) Light-modulated protein–protein interaction assay used in this work. The Gal4 DNA-binding domain (DBD) was linked to BcWCL1 or its variants with LOV domain deletion (BcWCL1^LOV∆^), PAS domains deletion (BcWCL1^PAS∆^), or a protein region containing the LOV domain (BcWCL1^LOV^) as a single module. The LOV* versions of BcWCL1 and BcWCL1^PAS∆^ proteins carry the C414S mutation. Similarly, the Gal4 transactivation domain (AD) was tied to the BcWCL2 or its variant with PAS domain deletion (BcWCL2^PAS∆^). Abbreviations: DD, constant darkness; BL, constant blue light; BLP, blue-light pulse of 2 h duration; Luc, luciferase reporter gene; *5XGAL1*, synthetic *GAL1* promoter.

To analyze the interaction between the components of the BcWCC, the original FUN-LOV optogenetic switch system (Figure 1A) developed in *S. cerevisiae* [32] was modified, as depicted in Figure 1B. Succinctly, the Gal4-DBD was fused to the full-length ORF of *bcwcl1* or its variants (see below), whereas the Gal4-AD was fused to the ORF of *bcwcl2* or its variant (Figure 1B). Therefore, protein–protein interactions between BcWCC components are expected to activate the luciferase reporter gene expression, much like the yeast two-hybrid system. Furthermore, to assess the effect of light on the protein–protein interaction, we performed the experiments under three different culture conditions: constant darkness (DD), constant blue light (BL), and a single blue-light pulse (BLP) of 2 h duration (Figure 1B). This experimental set-up can discriminate between protein–protein interactions that occur under constant culture conditions (e.g., DD and BL) and the effect of a BLP on the protein–protein interaction, the latter being detectable by the absence of luciferase expression in DD followed by Luc expression during the BLP.

**Figure 2:**
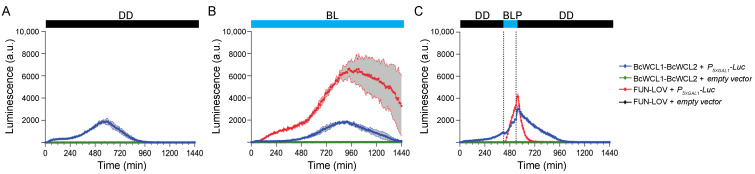
BcWCL1 and BcWCL2 proteins interact in the presence of blue light or its absence. Luciferase expression was measured as luminescence in arbitrary units (a.u.) of the yeast cell cultures. The protein–protein interaction activates luciferase expression controlled by the 5XGAL1 synthetic promoter (*P_5XGAL1_*) under three different experimental conditions: (**A**) constant darkness (DD), (**B**) constant blue light (BL), and (**C**) a single blue-light pulse (BLP) of 2 h duration (between dotted lines). In all panels, the FUN-LOV optogenetic switch [32] was used as a positive control of light-mediated protein–protein interaction that activates Luc expression. The average of luciferase expression determined in six biological replicates is shown, with the standard deviation represented as a shaded grey region.

As depicted in Figure 2, we measured the transcriptional activation of the luciferase reporter gene over a 24 h time window, observing luciferase expression and demonstrating the BcWCL1-BcWCL2 interaction in DD and BL (Figure 2A,B, respectively; and complete data set in Supplementary Figure S1). This observation agrees with a previously reported physical interaction between the mentioned proteins in the nucleus of *B. cinerea* [26]. The results also showed a time-shift in the peak of maximal luciferase expression in BL compared to DD (Figure 2A,B, respectively), which is due to growth kinetics differences in these conditions (a delay in the growth curve was observed in BL compared to DD; Supplementary Figure S1). Importantly, the FUN-LOV optogenetic system was used as a positive control of the light-mediated protein–protein interaction, observing a lack of transcriptional activation for the luciferase reporter gene in DD, Luc expression upon constant BL illumination, and a sharp and transient transcriptional activation after a single blue-light pulse (BLP) of 2 h duration (Figure 2), as previously demonstrated [32]. It is tempting to speculate that the 2 h BLP also led to a subtle increment in Luc expression in the BcWCL1-BcWCL2 interaction, observed as a shift in the trajectory of the blue curve depicted in Figure 2C. Indeed, Luc expression declined immediately after the BLP (during the second lights-off period) despite the yeast’s active growth (Supplementary Figure S1). However, we cannot conclude that the BcWCL1-BcWCL2 interaction responds to the BLP (Figure 2C and full data set in Supplementary Figure S1) since the BcWCL1-BcWCL2 interaction occurs in DD (Figure 2A). Thus, we cannot infer a possible effect of light in the BcWCL1-BcWCL2 interaction because luciferase expression and, thereby, the protein–protein interaction is observed in all the assayed conditions (Figure 2). Importantly, as a negative control of the protein–protein interaction experiments, we observed that single components containing only the full-length BcWCL1 or BcWCL2 proteins did not activate the luciferase reporter gene, showing only background expression irrespective of the culture condition (Supplementary Figure S2). Altogether, the results showed that full-length versions of BcWCL1 and BCWCL2 proteins can interact in the presence or absence of light.

### 3.2. Different Domains Participate in the Protein–Protein Interaction between BcWCL1 and BcWCL2

To uncover the protein domains that participate in the BcWCL1-BcWCL2 protein–protein interaction, careful *in silico* examination of the previously identified BcWCC was performed. According to the InterPro Scan analysis, the position of each predicted domain in BcWCL1 and BcWCL2 was localized, including their corresponding DNA binding domains (Table 1). For comparative purposes, we also include the *N. crassa* counterparts, as well as VVD, a LOV-containing photoresponse modulator widely studied in this fungus [44,45]. The GATA-type zinc finger domains of BcWCL1 and BcWCL2 are located at the C-terminal half of each TF (Table 1). The photoactive Cys residue for BcWCL1 was determined within the LOV domain after Clustal Omega alignment, employing, as references, the LOV-containing proteins WC-1 and VVD from *N. crassa*, and PHOT1 from *Arabidopsis thaliana*, whose photoactive residues have been experimentally validated (Table 1 and Supplementary Figure S4) [36,37,46].

Considering the location of the protein domains of interest in BcWCL1 and BcWCL2, we generated different mutant versions of these proteins, including: a deletion of the LOV domain (BcWCL1^LOV∆^; from aa 375–493), a deletion of the PAS domains (BcWCL1^PAS∆^; from aa 571–791), and a version of the protein containing only the LOV domain (BcWCL1^LOV^; see Table 1). Similarly, we generated a version of BcWCL2 with a deletion of the PAS domain (BcWCL2^PAS∆^; see Table 1). Therefore, we replaced the full-length BcWCL1 and BcWCL2 proteins with their different versions in the experimental setup (Figure 1B) to assay protein–protein interactions and the effects of light on them. 

**Figure 3:**
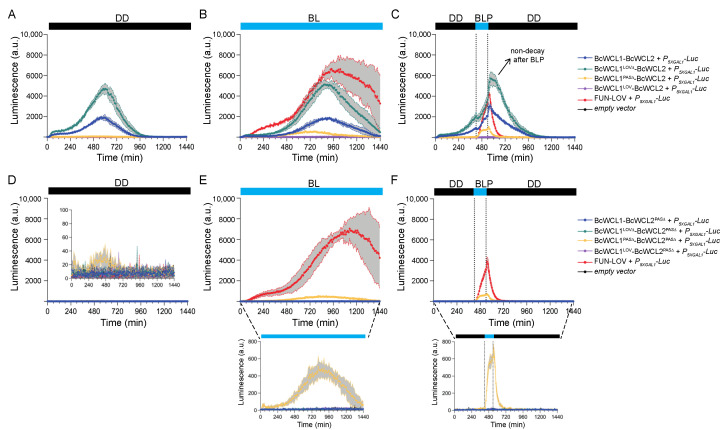
Protein–protein interaction between BcWCL1 and BcWCL2 is mediated mainly by PAS domains. Different variants of the BcWCL1 protein were assayed for protein–protein interaction with the full length BcWCL2 or BcWCL2 without the PAS domain. Luciferase expression was measured as luminescence in arbitrary units (a.u.) of the yeast cell cultures. The protein–protein interaction activates luciferase expression controlled by the 5XGAL1 synthetic promoter (*P_5XGAL1_*) under three different experimental conditions: (**A**,**D**) constant darkness (DD), (**B**,**E**) constant blue light (BL), and (**C**,**F**) a single blue-light pulse of 2 h duration (between dotted lines). The FUN-LOV optogenetic switch [32] was used as positive control of light-mediated protein–protein interaction that activates luciferase expression. In panels (**D**–**F**), the zoom shows the luciferase expression at lower scale. In all panels, the average of six biological replicates is shown, with standard deviation represented as shadowed grey regions.

When assessing the BcWCL1^LOV∆^-BcWCL2 interaction, we observed a protein–protein interaction in DD, BL, and BLP conditions, showing a higher luciferase expression compared to the BcWCL1-BcWCL2 interaction (Figure 3A–C; complete data set in Supplementary Figure S1). This result suggests that the LOV domain of BcWCL1 is a negative modulator of the BcWCL1-BcWCL2 interaction since its deletion augments the luciferase expression and, therefore, the strength of the protein–protein interaction, a phenomenon not observed in *N. crassa* for the interaction between WC-1 and WC-2 [22,47]. However, since the BcWCL1^LOV∆^-BcWCL2 interaction occurred in both DD and BL conditions (Figure 3A,B), we cannot infer whether this interaction responds to the BLP (Figure 3C). Indeed, and as noticed above, Luc expression declined after the BLP for the BcWCL1-BcWCL2 interacting pair and in the FUN-LOV system, but not in the case of the BcWCL1^LOV∆^-BcWCL2 interaction, observing a steady level of luminescence after the BLP (Figure 3C) and suggesting that the BcWCL1^LOV∆^-BcWCL2 interaction continues in the absence of blue light. 

Then, we assessed the BcWCL1^PAS∆^-BcWCL2 interaction, observing no protein–protein interaction in DD (Figure 3A and full data set in Supplementary Figure S1). Surprisingly, the luciferase expression was restored under BL, but with lower levels compared to the full-length proteins interaction BcWCL1-BcWCL2 (Figure 3B; full data set in Supplementary Figure S1). Notably, the BLP led to a transient reporter gene transcriptional activation observed only during the 2 h of illumination (Figure 3C; full data set in Supplementary Figure S1). These results indicate that the BcWCL1^PAS∆^-BcWCL2 interaction responds to blue light, suggesting that the LOV domain of BcWCL1 is necessary for light sensing and that an unidentified protein region participates in the interaction with BcWCL2. To discard an unlikely LOV-mediated interaction, we generated a BcWCL1 version including only the LOV domain (BcWCL1^LOV^; see Table 1), assessing its interaction with BcWCL2 under the same experimental conditions (Figure 1B). As expected, the BcWCL1^LOV^ was unable to interact with BcWCL2 as a single protein module in any of the culture conditions assayed (Figure 3A–C, and Supplementary Figure S1). Importantly, these results also suggest that an unidentified protein region of BcWCL1 participates in the protein interaction with BcWCL2 upon blue-light stimulation. In conclusion, the PAS domains of BcWCL1 are fundamental for its interaction with BcWCL2 in DD and BL conditions. However, in the absence of PAS domains (BcWCL1^PAS∆^), this protein responds to blue light potentially through its LOV domain, and where a protein region—but not the LOV domain itself—is involved in the protein–protein interaction with BcWCL2.

Finally, we assessed the contribution of the PAS domain of BcWCL2 on the protein–protein interaction with BcWCL1. Thus, the BcWCL2 version carrying a deletion in the PAS domain (BcWCL2^PAS∆^) was assessed for a protein–protein interaction with different variants of BcWCL1 (Figure 1B). As expected, BcWCL2^PAS∆^ completely abolished the interaction with BcWCL1 in all the illumination conditions assayed (Figure 3D–F; full data set in Supplementary Figure S3), showing that the BcWCL2 PAS domain is necessary for the protein–protein interaction. Surprisingly, we observed luciferase expression and, therefore, a protein–protein interaction between BcWCL1^PAS∆^ and BcWCL2^PAS∆^ under BL and BLP (Figure 3E,F; full data set in Supplementary Figure S3), confirming that blue light modulates their interaction and that additional yet unidentified regions in both proteins may serve as interacting domains. In conclusion, the PAS domains of BcWCL1 and BcWCL2 are principal contributors for the interaction of the BcWCC in the presence or absence of light. However, in the absence of PAS domains in both proteins, their light-mediated interaction seems to be conducted by an unidentified protein region, where the LOV domain of BcWCL1 should be necessary for light sensing, in a similar fashion to WC-1 and VVD photoreceptors from *N. crassa* [36,45]. 

### 3.3. The BcWCL1^PAS∆^ Protein Responds to Blue-Light Stimulation and Interacts with BcWCL2 or BcWCL2^PAS∆^

**Figure 4:**
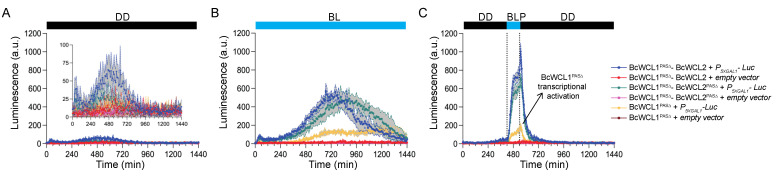
The protein interactions between BcWCL1^PAS∆^ and BcWCL2 or BcWCL2^PAS∆^ respond to blue-light stimulation. The BcWCL1 protein carrying a deletion in the PAS domains (BcWCL1^PAS∆^) was assayed for protein–protein interaction with the full length BcWCL2 or its variant without PAS domain (BcWCL2^PAS∆^), respectively. The individual contribution of BcWCL1^PAS∆^ in the light-mediated transcriptional activation was included as control. Luciferase expression was measured as luminescence in arbitrary units (a.u.) of the yeast cell cultures. The protein–protein interaction activates luciferase expression controlled by the 5XGAL1 synthetic promoter (*P_5XGAL1_*) under three different conditions: (**A**) constant darkness (DD), (**B**) constant blue light (BL), and (**C**) a single blue-light pulse of 2 h duration (between dotted lines). In all panels, the average of six biological replicates is shown, with standard deviation represented as shadowed regions.

BcWCL1 and BcWCL2 are TFs that may recruit the transcriptional machinery in yeast, potentially activating the reporter gene transcription without the necessity of an interacting partner. This possibility prompted us to assess the individual contribution of BcWCL1, BcWCL2, and its protein variants in the readout detected by the experimental setup. Therefore, we generated yeast strains carrying a single plasmid encoding BcWCL1 or BcWCL2 protein variants but not containing the plasmid encoding the interacting partner (Supplementary Figure S2). In general, we observed luciferase expression only for BcWCL1^PAS∆^ in BL or upon BLP, suggesting a light-triggered conformational change in BcWCL1^PAS∆^ that promotes transcription (Figure 4 and Supplementary Figure S2), similar to that observed in the WC-1 protein of *N. crassa* [36]. We estimated the contribution of BcWCL1^PAS∆^ as a single component in our dataset, comparing the luciferase expression in all of the combinations that include this protein version. As depicted in Figure 4, the individual contribution of BcWCL1^PAS∆^ transcriptional activation corresponds to 23% and 31% of the signal detected in the BcWCL1^PAS∆^-BcWCL2 and BcWCL1^PAS∆^-BcWCL2^PAS∆^ interaction, respectively, which was estimated as the area under the curve for the luciferase expression signal under BLP stimulation (Figure 4C). In the aggregate, these results confirm that BcWCL1^PAS∆^ indeed interacts with BcWCL2 and BcWCL2^PAS∆^ upon blue-light stimulation (Figure 4), reinforcing the idea that a protein region including the LOV domain of BcWCL1 modulates the interaction with BcWCL2 depending on the illumination conditions. 

**Figure 5:**
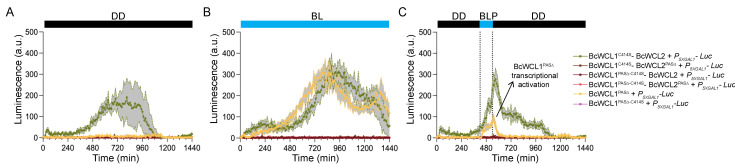
The BcWCL1 and BcWCL1^PAS∆^ proteins are capable of blue-light sensing. The BcWCL1 protein carrying the C414S mutation (BcWCL1^C414S^) or its version without PAS domains and containing the C414S mutation (BcWCL1^PAS∆-C414S^) were assayed for protein–protein interaction with the full length BcWCL2 or its variant without PAS domain (BcWCL2^PAS∆^), respectively. The individual contribution of BcWCL1^PAS∆^ in the light-mediated transcriptional activation was included as control. Luciferase expression was measured as luminescence in arbitrary units (a.u.) of the yeast cell cultures. The protein–protein interaction activates luciferase expression controlled by the 5XGAL1 synthetic promoter (*P_5XGAL1_*) under three different conditions: (**A**) constant darkness (DD), (**B**) constant blue light (BL), and (**C**) a single blue-light pulse of 2 h duration (between dotted lines). In all panels, the average of six biological replicates is shown, with standard deviation represented as shadowed regions.

Finally, we sought to confirm that BcWCL1 and BcWCL1^PAS∆^ are capable of blue-light sensing by introducing a point mutation in the photoactive cysteine within the LOV domain (Table 1), replacing this amino acid with serine (C414S). Thus, we generated the BcWCL1^C414S^ and BcWCL1^PAS∆-C414S^ protein versions, which were assayed for protein–protein interactions using the same experimental set-up depicted in Figure 1B. The results demonstrated that BcWCL1^C414S^ is still able to interact with BcWCL2 in all of the assayed conditions (Figure 5; full data set in Supplementary Figure S5). However, the BcWCL1^C414S^-BcWCL2 interaction showed a lower strength than the wild-type version of these proteins (compare Figure 2 and Figure 5), supporting the idea that the LOV domain of BcWCL1 modulates the protein interaction with BcWCL2. Furthermore, the BcWCL1^C414S^-BcWCL2 interaction also showed a different behavior compared to the BcWCL1^LOV∆^-BcWCL2 interaction (compare Figure 3 and Figure 5), which is probably due to different effects on the BcWCL1 protein structure causes by LOV domain deletion compared to the point mutation (C414S). Despite this observation, both versions of BcWCL1 (BcWCL1^LOV∆^ and BcWCL1^C414S^) retain the capacity to interact with BcWCL2 and lose the blue-light response, confirming the importance of the LOV domain in BcWCL1 light sensing. Importantly, the BcWCL1^C414S^-BcWCL2 interaction was primarily mediated by PAS domains since the deletion of this domain in BcWCL2 (BcWCL2^PAS∆^) completely abolished the protein–protein interaction (Figure 5). Interestingly, when we assessed the effect of the C414S mutation in the BcWCL1^PAS∆^ protein context (BcWCL1^PAS∆-C414S^), the light-response of this protein was entirely disrupted (Figure 5; full data set in Supplementary Figure S5). In addition, the BcWCL1^PAS∆-C414S^ protein was unable to interact under light-mediated conditions with BcWCL2 or BcWCL2^PAS∆^ and lost its individual light-triggered transcriptional activation (Figure 5). Therefore, the results prove that BcWCL1 and BcWCL1^PAS∆^ can perceive blue light through their LOV domains. 

## 4. Discussion

Herein, we provide proof that the BcWCL1 of *B. cinerea* can sense blue light. Eleven photoreceptors are encoded in the *B. cinerea* genome, but, besides the blue-light receptors BcVVD1 [48] and BcLOV4 [49], no biophysical information is available that shows that the molecular function of any of these proteins can be modulated by a specific light wavelength. Therefore, BcWCL1 is the third. Importantly, as mentioned above, early investigations have provided substantial proof of broad-spectrum light detection capacities displayed by *B. cinerea* [10,11,12,13,14]. Nonetheless, no biochemical (e.g., FAD-binding) or photochemical evidence of light responses have proved that BcWCL1 can sense blue light. Regardless, BcWCL1 light-dependent responses have been determined [5,17], including the light inducibility of *bcfrq1*, the central pacemaker of the *B. cinerea* circadian clock [27,28]. As demonstrated herein, a relatively simple modification of the previously described FUN-LOV optogenetic switch [32] provides an excellent and orthogonal biological system in which to assess protein–protein interactions when at least one of the interacting partners is a photoreceptor (Figure 1). This highlights the relevance of a blind system that can be subjected to different light intensities and qualities [50]. Importantly, the luciferase reporter gene in *S. cerevisiae* allowed us to detect subtle variations in the dynamics of the protein–protein interactions, such as those observed under BLP. Likewise, the interaction between BcWCL1 and BcWCL2 (both in light and darkness) was observed as the activation of this reporter gene (Figure 2).

Interestingly, both in *N. crassa* and *B. cinerea,* early and late transcriptional responses to light have been reported [51,52], which is explained by a transcriptional cascade involving several light-induced transcription factors [53], a phenomenon that has also begun to be deciphered in *B. cinerea* through the use of loss-of-function mutants [52,54], as well as through recent systems biology approaches [55]. In addition, photosensor proteins seem to have dark-related functions, as recently highlighted [4], and, as a matter of fact, BcWCL1 is needed to inhibit conidia development in the dark [17]. Furthermore, we demonstrate that, as expected, the protein–protein interaction between BcWCL1 and BcWCL2 is mainly through their PAS domains, where the deletion of the PAS domains in BcWCL1 or BcWCL2 severally impairs the protein–protein interaction in all the conditions assayed (Figure 3). The BcWCL1-BcWCL2 interaction was previously reported in *B. cinerea* [26]. However, we demonstrated that PAS domains are indeed necessary for this protein–protein interaction. 

Interestingly, the deletion of the PAS domains from BcWCL1 (BcWCL1^PAS∆^) revealed the protein’s responsiveness to light and its blue-light-dependent interaction with BcWCL2 or BcWCL2^PAS∆^. The BcWCL1^PAS∆^ protein was able to activate the reporter gene without the need for an interacting partner, suggesting a possible light-triggered conformational change in BcWCL1^PAS∆^ that exposes its activation domain. This result is not unexpected since BcWCL1 is a photoreceptor and transcription factor in *B. cinerea* [5,17,26], which could be capable of transcriptional activation in yeast. Furthermore, the WC-1 protein of *N. crassa* is capable of self-dimerization through a LOV-LOV interaction [56], which could also occur in BcWCL1 and BcWCL1^PAS∆^ proteins. However, further experiments are required to uncover this phenomenon. Finally, we performed a point mutation in the photoactive cysteine (C414S) of BcWCL1 and BcWCL1^PAS∆^, demonstrating that these proteins are capable of blue-light sensing through their LOV domain. Importantly, the results presented here for BcWCL1-BcWCL2 are based on a yeast protein–protein interaction assay, which may not necessarily represent the behavior of these proteins in the circadian context of *B. cinerea*.

Overall, these results illustrate the potential of fungal photoreceptors as a relatively underexplored source of building blocks to construct novel optogenetic switches for synthetic biology approaches, enabling light-controlled gene expression in yeast and many other biological systems [57]. In this sense, the activation/deactivation kinetics of BcWCL1^PAS∆^ upon a single BLP is similar to the FUN-LOV switch (Figure 3F), supporting the future application of BcWCL1^PAS∆^ in the development of novel optogenetic switches.

As has recently become apparent in the field of photobiology [3], there is a need to understand how distinct photoreceptors interact with each other to explain the complexities of photobiological responses observed in, for instance, fungal systems. In this context, future work will provide evidence of red/blue light receptor interactions in *B. cinerea*, whose genome encodes three PAS-containing phytochromes and enables the validation of an old “Two-receptor-model” in which red/blue photoreceptors interact in this organism [12]. Altogether, the work presented herein highlights the peculiarities of *B. cinerea* photobiology and the utility of a modified blind assay in yeast cells, allowing us to determine protein–protein interactions when one of the interacting partners is a light receptor protein. Hopefully, in the near future, this system will allow us to determine protein interactions between different fungal photoreceptors in the presence or absence of light.

## 5. Conclusions

In conclusion, BcWCL1 and BcWCL2 interact in the presence or absence of light, primarily through their PAS domains. The deletion of the PAS domains in the BcWCL1 protein (BcWCL1^PAS∆^) unmasks the light response of this protein, which interacts with both BcWCL2 and BcWCL2^PAS∆^ upon blue-light stimulation. These protein–protein interactions, under illumination conditions, occur through an unidentified protein region and where the LOV domain of BcWCL1 is necessary for the light response. Finally, the BcWCL1^PAS∆^ protein is capable of blue-light sensing through its LOV domain since the mutation C414S completely disrupted its light-mediated transcriptional activation and interaction with BcWCL2 or BcWCL2^PAS∆^.

## Figures and Tables

**Table 1:** Position of the protein domains in BcWCL1 and BcWCL2 of *B. cinerea* and its orthologous in *N. crassa*. The position of each domain was predicted using the InterPro Scan tool [34]. The cysteine (Cys) position within the LOV domain was obtained through a protein alignment. For comparative purposes, the NcVVD and PHOT1 photoreceptors of *N. crassa* and *A. thaliana*, respectively, were included.

Protein	Gene ID	Protein Length (aa)	DNA Binding Domain (aa)	LOV Domain (aa)	LOV Domain Cys (aa)	PAS Domain (aa)
BcWCL1	Bcin02g07400	1137	932-984	375–493	414	571–670; 697–791
BcWCL2	Bcin05g05530	509	448–500	-	-	146–244
NcWC-1	NCU02356	1167	928–987	389–505	428	585–684; 705–800
NcWC-2	NCU00902	530	462–514	-	-	162–255
NcVVD	NCU03967	186	-	73–182	108	-
PHOT1	AT3G45780	996	-	485–577 (LOV2)	512	-

## Data Availability

The datasets supporting reported results are available upon request to the corresponding author.

## Supplementary Materials

The following are available online at https://www.mdpi.com/article/10.3390/jof8050486/s1, Figure S1: Raw data for the protein–protein interaction assay using BcWCL1 or its protein variants versus BcWCL2. Figure S2: Raw data for the single components assay using BcWCL1, BcWCL2, or its protein variants. Figure S3: Raw data for the protein–protein interaction assay using BcWCL1 or its protein variants versus BcWCL2^PAS∆^. Figure S4: Alignment of LOV domains from *N. crassa* and *B. cinerea* proteins (NcWC-1, NcVVD, BcWCL1, and BcVVD1). Figure S5: Raw data for the protein–protein interaction assay using BcWCL1^C414S^ or its protein variant without PAS domain (BcWCL1^PAS∆-C414S^) versus BcWCL2 or BcWCL2^PAS∆^. Table S1: Primers used in this work, Table S2: Plasmids generated in this work, Table S3: *S. cerevisiae* strains used and generated in this work.
