# Supplementary material for: Interactions between Core Elements of the Botrytis cinerea Circadian Clock Are Modulated by Light and Different Protein Domains
Source: J Fungi (Basel). 2022 May 6;8(5):486. doi: 10.3390/jof8050486 (PMC9144814; doi:10.3390/jof8050486)
Supplement: Supplementary file 1 [file jof-08-00486-s001.zip › jof-1663209-supplementary.pdf]

Supplementary Information

Interactions between core elements of the *Botrytis cinerea* circadian clock are modulated by light and different protein domains

Vicente Rojas[1,2§], Francisco Salinas[2,3§], Andrés Romero[2,3], Luis F. Larrondo[1,2] and Paulo Canessa[2,4*]

1 Departamento de Genética Molecular y Microbiología, Facultad de Ciencias Biológicas, Pontificia Universidad Católica de Chile, Santiago, postal code 8331150, Chile; vlrojas@uc.cl (V.R.); llarrondo@bio.puc.cl (L.F.L)

2 ANID–Millennium Science Initiative–Millennium Institute for Integrative Biology (iBIO), Santiago, postal code 8331150, Chile.

3 Instituto de Bioquímica y Microbiología, Facultad de Ciencias, Universidad Austral de Chile, Valdivia, postal code 5090000, Chile; francisco.salinas@uach.cl

4 Centro de Biotecnologia Vegetal, Facultad de Ciencias de la Vida, Universidad Andres Bello, Santiago, postal code 8370186, Chile; paulo.canessa@unab.cl

* Correspondence: paulo.canessa@unab.cl

§ These authors contributed equally to this work.

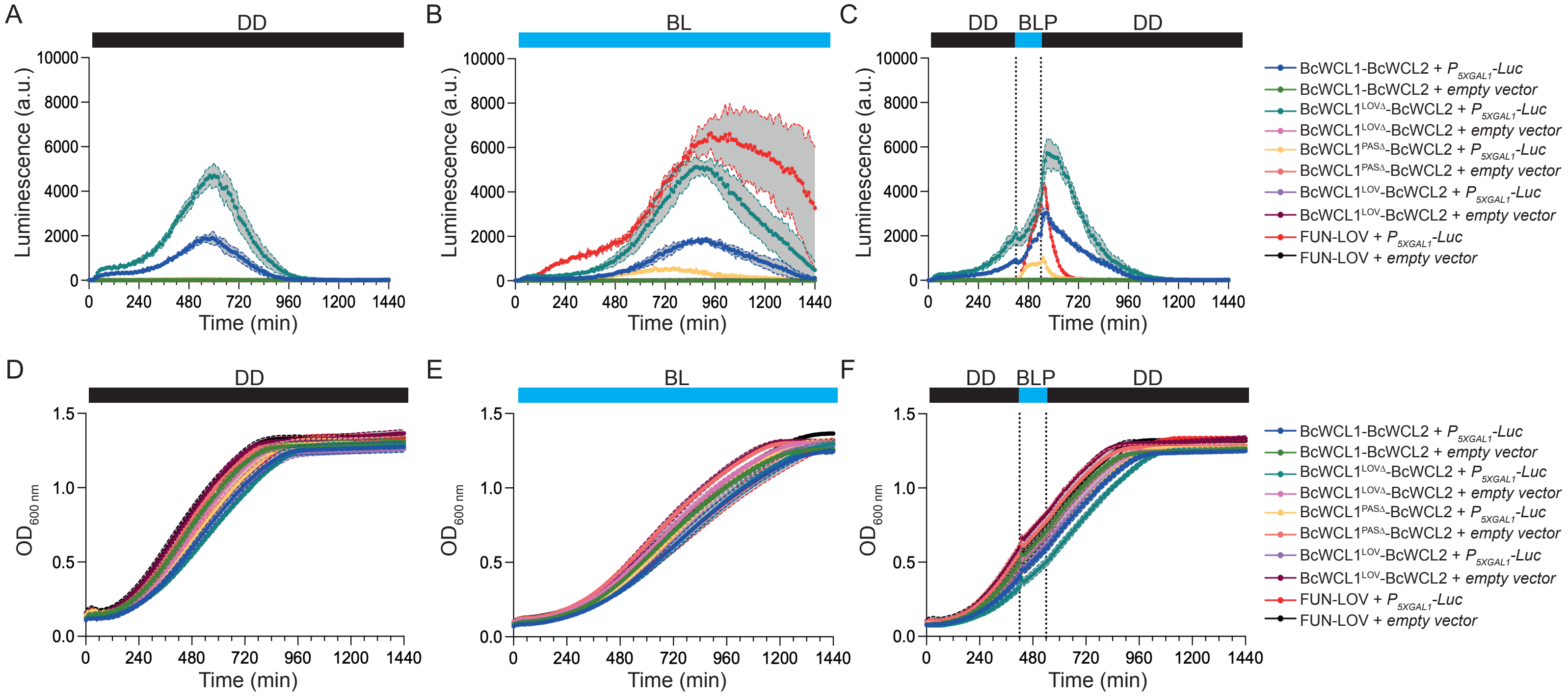

Supplementary Figure S1. Raw data for the protein-protein interaction assay using BcWCL1 or its protein variants versus BcWCL2. The luciferase expression (panels **A**, **B** and **C**) measured as luminescence in arbitrary units (a.u.) and the Optical Density (OD) at 600 nm (panels **D**, **E**, and **F**) of the yeast cultures are shown. The protein-protein interaction activates luciferase expression controlled by the 5XGAL1 synthetic promoter (P_{5XGAL1}) under three different experimental conditions: (**A** and **D**) constant darkness (DD), (**B** and **E**) constant blue-light (BL), and (**C** and **F**) a single blue-light pulse (BLP) of 2h (dotted lines). In all panels, the average of six biological replicates is shown, with the standard deviation represented as a shaded region.

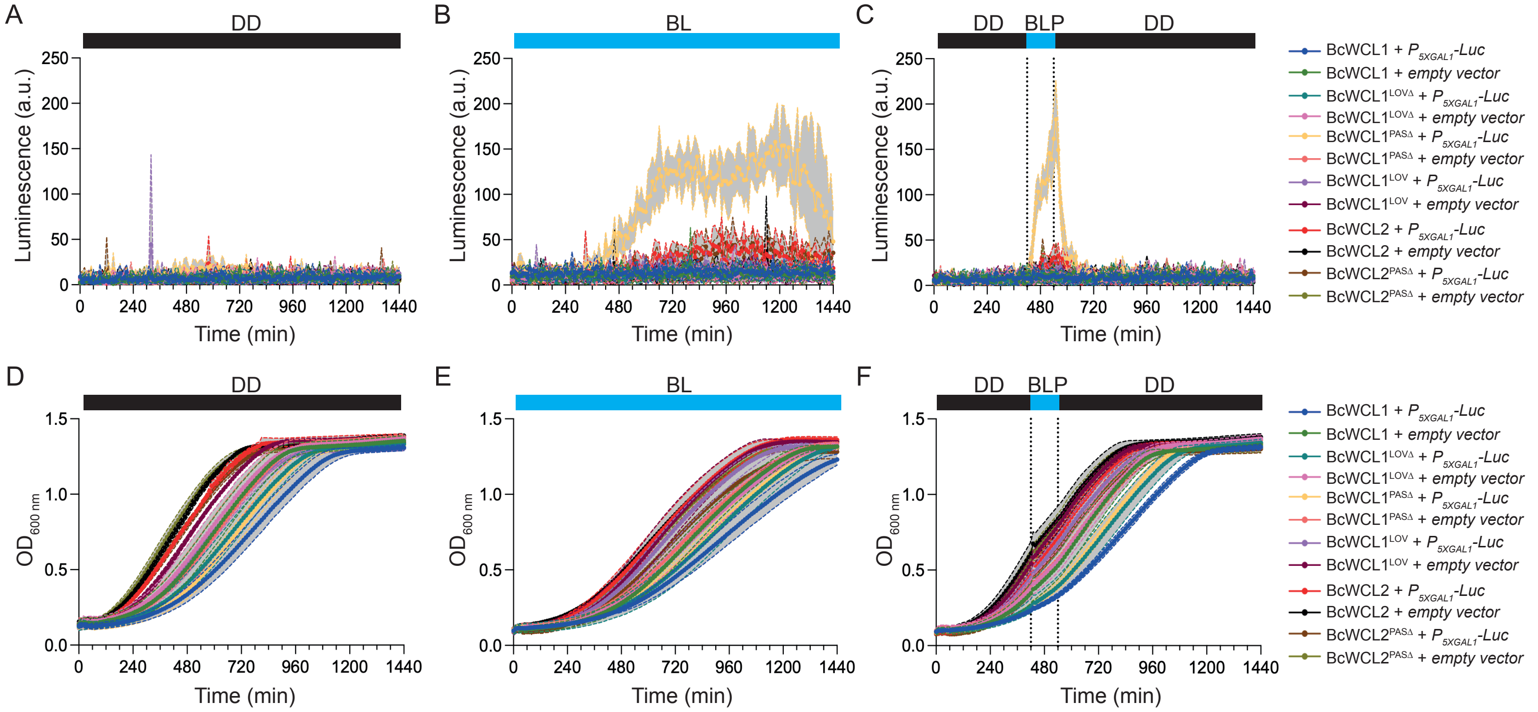

Supplementary Figure S2. Raw data for the single components assay using BcWCL1, BcWCL2, or its protein variants. The luciferase expression (panels **A**, **B** and **C**) measured as luminescence in arbitrary units (a.u.) and the Optical Density (OD) at 600 nm (panels **D**, **E**, and **F**) of the yeast cultures is shown. The protein-protein interaction activates luciferase expression controlled by the 5XGAL1 synthetic promoter (*P_{5XGAL1}*) under three different experimental conditions: (**A** and **D**) constant darkness (DD), (**B** and **E**) constant blue-light (BL), and (**C** and **F**) a single blue-light pulse (BLP) of 2h (dotted lines). In all panels, the average of six biological replicates is shown, with the standard deviation represented as a shaded region.

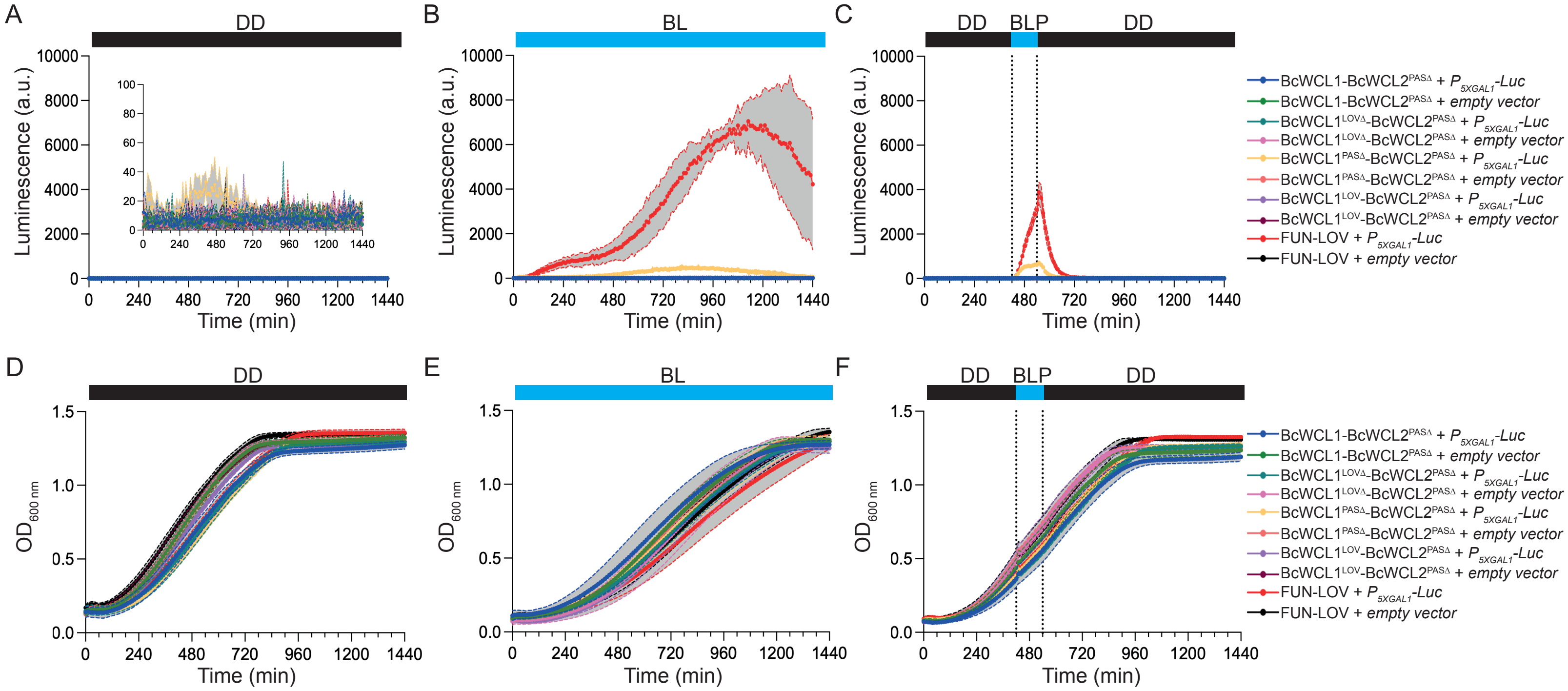

Supplementary Figure S3. Raw data for the protein-protein interaction assay using BcWCL1 or its protein variants versus BcWCL2$^{PAS\Delta}$. The luciferase expression (panels **A**, **B** and **C**) measured as luminescence in arbitrary units (a.u.) and the Optical Density (OD) at 600 nm (panels **D**, **E**, and **F**) of the yeast cultures are shown. The protein-protein interaction activates luciferase expression controlled by the 5XGAL1 synthetic promoter (P_{5XGAL1}) under three different experimental conditions: (**A** and **D**) constant darkness (DD), (**B** and **E**) constant blue-light (BL), and (**C** and **F**) a single blue-light pulse (BLP) of 2h (dotted lines). In the panels, the average of six biological replicates is shown, with the standard deviation represented as a shaded region.

Supplementary Figure S4: Alignment of LOV domains from *N. crassa* and *B. cinerea* proteins (NcWC-1, NcVVD, BcWCL1, and BcVVD1). Black residues are identical in all LOV domains. The most conserved region among LOV domains. The LOV domain of the PHOT1 photoreceptor from *A. thaliana* was included as control. The red arrow depicts the photoactive cysteine.

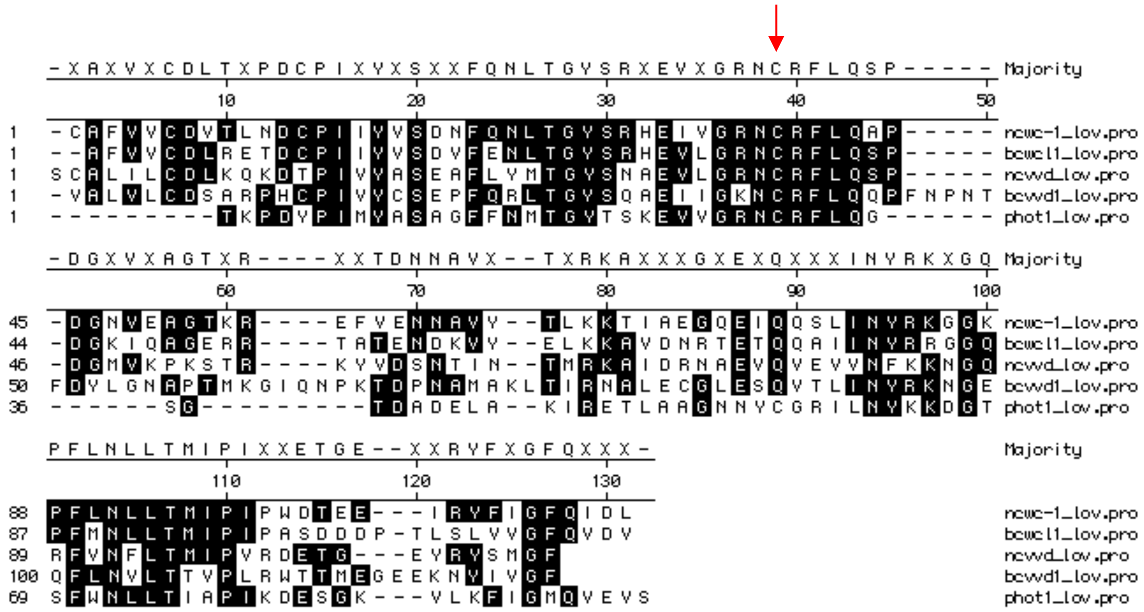

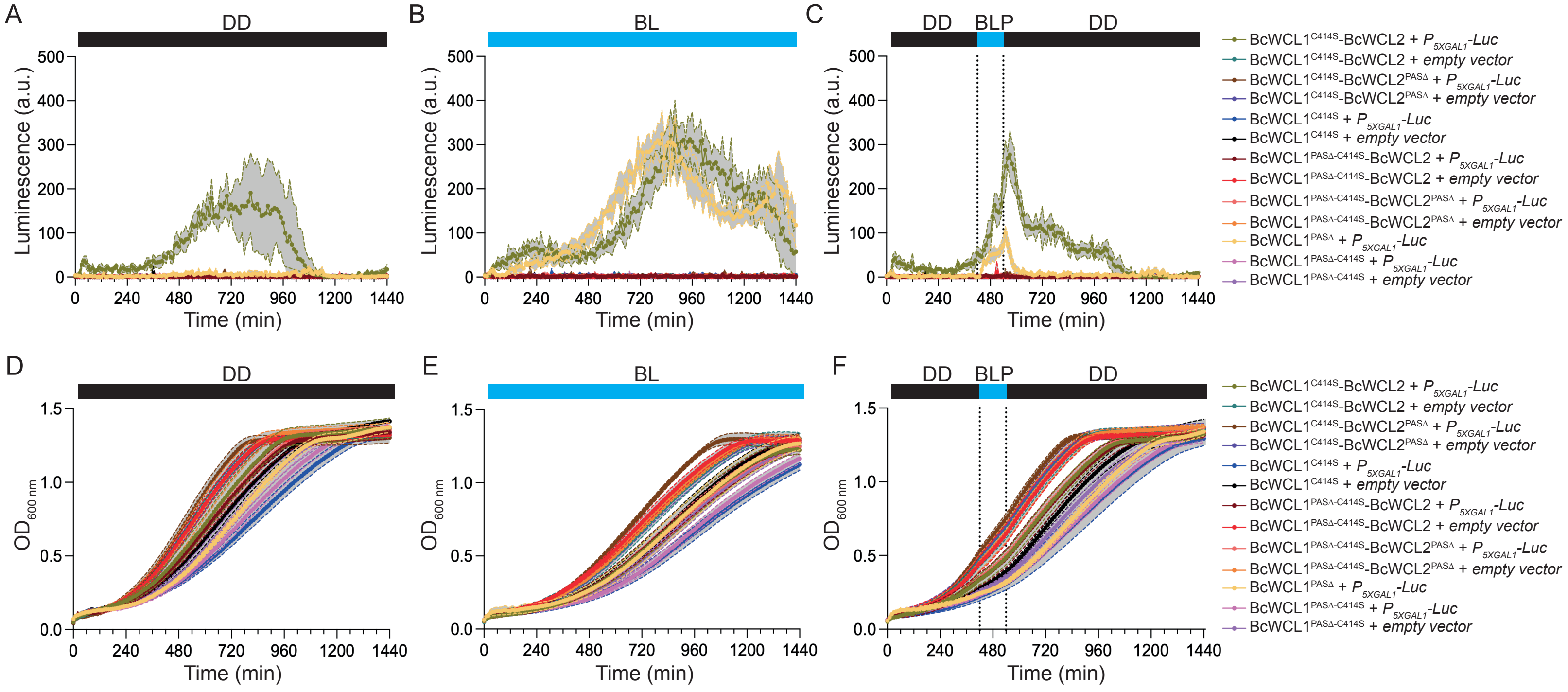

Supplementary Figure S5. Raw data for the protein-protein interaction assay using BcWCL1^{C414S} or its protein variant without PAS domain (BcWCL1$^{PASΔ-C414S}$) versus BcWCL2 or BcWCL2^{PASΔ}. The luciferase expression (panels **A**, **B** and **C**) measured as luminescence in arbitrary units (a.u.) and the Optical Density (OD) at 600 nm (panels **D**, **E**, and **F**) of the yeast cultures are shown. The protein-protein interaction activates luciferase expression controlled by the 5XGAL1 synthetic promoter (P_{5XGAL1}) under three different experimental conditions: (**A** and **D**) constant darkness (DD), (**B** and **E**) constant blue-light (BL), and (**C** and **F**) a single blue-light pulse (BLP) of 2h (between dotted lines). In the panels, the average of six biological replicates is shown, with the standard deviation represented as a shaded region.

Supplementary Table S1. Primers used in this work.

Name	Sequence (5’-3’)	Length (nt)	Orientation	Description
oL3758	AGCGGATAACAATTTCACACAGGAAACAGCTAGG CGCATGCAACTTCTTT	50	Fw	Recombination of P_{ADH1} with pRS423 or pRS425
oL3586	CATATGTATATGAGATAGTT	20	Rv	P_{ADH1} amplification
oL3587	GCATACAATCAACTATCTCATATACATATGATGCCA ATGACCCAAGCAGA	50	Fw	P_{ADH1} - BcWCL1 recombination
oL3590	TTCGATAGAAGACAGTAGCTTCATGGATCCATTCA TTCCACTCGTTTTCC	50	Rv	Gal4DBD- BcWCL1 recombination
oL3591	GGATCCATGAAGCTACTGTC	20	Fw	Gal4DBD amplification
oL3148	GGTAACGCCAGGGTTTTCCCAGTCACGACGGCCG GTAGAGGTGTGGTCAA	50	Rv	$ADH2_{ter}$ recombination with pRS423 or pRS425
oL3592	GCATACAATCAACTATCTCATATACATATGATGTCA GAGGGGGATACATC	50	FW	P_{ADH1} – BcWCL2 recombination
oL3593	CTCGGGAATTAATTCCGCTTTATCGGATCCAGAGC CTGGGGAAGGTCCCG	50	Rv	Gal4AD- BcWCL2 recombination
oL3594	GGATCCGATAAAGCGGAATT	20	Fw	Gal4AD amplification
oL3598	CTCGGGAATTAATTCCGCTTTATCGGATCCAGCGG AACGGTGCCTGTACA	50	Rv	Gal4AD- BcWCL2 recombination
oL3947	GCATACAATCAACTATCTCATATACATATGACGAAC ATATATTCCAGCAC	50	Fw	P_{ADH1} - BcWCL1^{LOV} recombination
oL3948	TTCGATAGAAGACAGTAGCTTCATGGATCCGCCTT GTTTATAATTCATCG	50	Rv	Gal4DBD- BcWCL1^{LOV} recombination
oL3588	ATTTCTTCCCAATACCTCATGTC	23	Rv	Internal BcWCL1
oL3995	AACAGCTCAGTCAATGATTTCACGAGACGAAGACC CTCGCCAACAGCAGC	50	Fw	BcWCL2$^{PAS\Delta}$ amplification
oL3945	TCGTCTCGTGAAATCATTGA	20	Rv	Internal BcWCL2
oL3588	ATTTCTTCCCAATACCTCATGTC	23	Rv	Internal BcWCL1
oL3589	TATTCAAGACATGAGGTATTGGGAAGAAATTCTCG ATTTCTCCAATCACCAGATG	55	Fw	Internal BcWCL1 C414S mutation

Supplementary Table S2. Plasmids generated in this work.

Plasmid number	Name	Genetic construct	Vector backbone	Reference
1	BcWCL1	P_{ADH1}-*BcWCL1-GAL4 DBD-ADH2*$_{ter}$	pRS423	This work
2	BcWCL1$^{LOV\Delta}$	P_{ADH1}-*BcWCL1*$^{LOV\Delta}$-*GAL4 DBD-ADH2*$_{ter}$	pRS423	This work
3	BcWCL1$^{PAS\Delta}$	P_{ADH1}-*BcWCL1*$^{PAS\Delta}$-*GAL4 DBD-ADH2*$_{ter}$	pRS423	This work
4	BcWCL1^{LOV}	P_{ADH1}-*BcWCL1*^{LOV}-*GAL4 DBD-ADH2*$_{ter}$	pRS423	This work
5	BcWCL2	P_{ADH1}-*BcWCL2-GAL4 AD-ADH2*$_{ter}$	pRS425	This work
6	BcWCL2$^{PAS\Delta}$	P_{ADH1}-*BcWCL2*$^{PAS\Delta}$-*GAL4 AD-ADH2*$_{ter}$	pRS425	This work
7	BcWCL1^{C414S}	P_{ADH1}-*BcWCL1*^{C414S}-*GAL4 DBD-ADH2*$_{ter}$	pRS423	This work
8	BcWCL1$^{PAS\Delta\text{-}C414S}$	P_{ADH1}-*BcWCL1*$^{PAS\Delta\text{-}C414S}$-*GAL4 DBD-ADH2*$_{ter}$	pRS423	This work
9	5XPGAL1-LUC	*KanMxRV-*P_{5XGAL1}-*Luc-CYC1*$_{ter}$	pRS426	32
10	WC-1	P_{ADH1}-*WC-1 LOV-GAL4 BDB-ADH2*$_{ter}$	pRS423	32
11	VVD	P_{ADH1}-*VVD-GAL4 AD-ADH2*$_{ter}$	pRS425	32

Supplementary Table S3. *S. cerevisiae* strains used and developed in this work.

Strain Name	Genotype*	Source
BY4741	*MATa his3Δ1 leu2Δ0 met15Δ0 ura3Δ0*	Euroscarf
gal4Δ-gal80Δ	*BY4741; gal4Δ::NatMx gal80Δ::HphMx*	32
Y196	*gal4Δ-gal80Δ; plasmids 1, 5, and 9*	This work
Y202	*gal4Δ-gal80Δ; plasmids 1, 5, and pRS426*	This work
Y359	*gal4Δgal80Δ; plasmids 1, 6, and 9*	This work
Y360	*gal4Δgal80Δ; plasmids 1, 6, and pRS426*	This work
Y520	*gal4Δ-gal80Δ; plasmids 2, 5, and 9*	This work
Y521	*gal4Δ-gal80Δ; plasmids 2, 5, and pRS426*	This work
Y512	*gal4Δ-gal80Δ; plasmids 3, 5, and 9*	This work
Y513	*gal4Δ-gal80Δ; plasmids 3, 5, and pRS426*	This work
Y516	*gal4Δ-gal80Δ; plasmids 3, 6, and 9*	This work
Y517	*gal4Δ-gal80Δ; plasmids 3, 6, and pRS426*	This work
Y361	*gal4Δgal80Δ; plasmids 4, 5, and 9*	This work
Y362	*gal4Δgal80Δ; plasmids 4, 5, and pRS426*	This work
Y377	*gal4Δgal80Δ; plasmids 4, 6, and 9*	This work
Y378	*gal4Δgal80Δ; plasmids 4, 6, and pRS426*	This work
Y363	*gal4Δgal80Δ; plasmids 1 and 9*	This work
Y364	*gal4Δgal80Δ; plasmids 1 and pRS426*	This work
Y530	*gal4Δgal80Δ; plasmids 2 and 9*	This work
Y531	*gal4Δgal80Δ; plasmids 2 and pRS426*	This work
Y528	*gal4Δgal80Δ; plasmids 3 and 9*	This work
Y529	*gal4Δgal80Δ; plasmids 3 and pRS426*	This work
Y367	*gal4Δgal80Δ; plasmids 4 and 9*	This work
Y368	*gal4Δgal80Δ; plasmids 4 and pRS426*	This work
Y371	*gal4Δgal80Δ; plasmids 5 and 9*	This work
Y372	*gal4Δgal80Δ; plasmids 5 and pRS426*	This work
Y373	*gal4Δgal80Δ; plasmids 6 and 9*	This work
Y374	*gal4Δgal80Δ; plasmids 6 and pRS426*	This work
Y288	*gal4Δgal80Δ; plasmids 5, 7, and 9*	This work
Y289	*gal4Δgal80Δ; plasmids 5, 7, and pRS426*	This work
Y290	*gal4Δgal80Δ; plasmids 6, 7, and 9*	This work
Y291	*gal4Δgal80Δ; plasmids 6, 7, and pRS426*	This work
Y296	*gal4Δgal80Δ; plasmids 7 and 9*	This work
Y297	*gal4Δgal80Δ; plasmids 7 and pRS426*	This work
Y278	*gal4Δgal80Δ; plasmids 5, 8, and 9*	This work
Y279	*gal4Δgal80Δ; plasmids 5, 8, and pRS426*	This work
Y280	*gal4Δgal80Δ; plasmids 6, 8, and 9*	This work
Y281	*gal4Δgal80Δ; plasmids 6, 8, and pRS426*	This work
Y286	*gal4Δgal80Δ; plasmids 8 and 9*	This work
Y287	*gal4Δgal80Δ; plasmids 8 and pRS426*	This work
Y161	*gal4Δgal80Δ; plasmids 9, 10, and 11 (FUN-LOV)*	32
Y159	*gal4Δgal80Δ; plasmids 9, 10, and pRS426*	32

*Plasmids information in Supplementary Table S2
